# Somatostatin Primes Endothelial Cells for Agonist-Induced Hyperpermeability and Angiogenesis In Vitro

**DOI:** 10.3390/ijms23063098

**Published:** 2022-03-13

**Authors:** Muhammad Aslam, Hafiza Idrees, Peter Ferdinandy, Zsuzsanna Helyes, Christian Hamm, Rainer Schulz

**Affiliations:** 1 Experimental Cardiology, Department of Cardiology and Angiology, Justus Liebig University, Aulweg 129, 35392 Giessen, Germany; hafiza.idrees@innere.med.uni-giessen.de (H.I.); christian.hamm@innere.med.uni-giessen.de (C.H.); 2 Department of Cardiology, Kerckhoff Clinic GmbH, 61231 Bad Nauheim, Germany; 3 DZHK (German Centre for Cardiovascular Research), Partner Site Rhein-Main, 61231 Bad Nauheim, Germany; 4 Department of Pharmacology and Pharmacotherapy, Semmelweis University, 1089 Budapest, Hungary; peter.ferdinandy@pharmahungary.com; 5 Pharmahungary Group, 6722 Szeged, Hungary; 6 Department of Pharmacology and Pharmacotherapy, Medical School and Szentágothai Research Centre, University of Pécs, 7624 Pécs, Hungary; zsuzsanna.helyes@aok.pte.hu; 7 PharmInVivo Ltd., 7624 Pécs, Hungary; 8 Institute of Physiology, Justus Liebig University, 35392 Giessen, Germany; rainer.schulz@physiologie.med.uni-giessen.de

**Keywords:** somatostatin receptors, Akt, MAPK, angiogenesis, endothelial permeability, RhoA/Rock, MYPT1, cAMP

## Abstract

Somatostatin is an inhibitory peptide, which regulates the release of several hormones, and affects neurotransmission and cell proliferation via its five G_i_ protein-coupled receptors (SST_1-5_). Although its endocrine regulatory and anti-tumour effects have been thoroughly studied, little is known about its effect on the vascular system. The aim of the present study was to analyse the effects and potential mechanisms of somatostatin on endothelial barrier function. Cultured human umbilical vein endothelial cells (HUVECs) express mainly SST_1_ and SST_5_ receptors. Somatostatin did not affect the basal HUVEC permeability, but primed HUVEC monolayers for thrombin-induced hyperpermeability. Western blot data demonstrated that somatostatin activated the phosphoinositide 3-kinases (PI3K)/protein kinase B (Akt) and p42/44 mitogen-activated protein kinase (MAPK) pathways by phosphorylation. The HUVEC barrier destabilizing effects were abrogated by pre-treating HUVECs with mitogen-activated protein kinase kinase/extracellular signal regulated kinase (MEK/ERK), but not the Akt inhibitor. Moreover, somatostatin pre-treatment amplified vascular endothelial growth factor (VEGF)-induced angiogenesis (3D spheroid formation) in HUVECs. In conclusion, the data demonstrate that HUVECs under quiescence conditions express SST_1_ and SST_5_ receptors. Moreover, somatostatin primes HUVECs for thrombin-induced hyperpermeability mainly via the activation of MEK/ERK signalling and promotes HUVEC proliferation and angiogenesis in vitro.

## 1. Introduction

Somatostatin (SST), also known as the somatotropin-release inhibiting factor (SRIF), is a widely distributed peptide throughout the body. It exerts a broad range of biological activities, including the inhibition of growth hormone, thyroid-stimulating hormone, gastrointestinal hormone and neuropeptide release [1,2]. SST exists in 14 and 28 amino acid-containing forms (SST14 and SST28) derived from the same 92-amino acid prosomatostatin [1,3] that activates the same receptors. SST14 is predominantly expressed in the central nervous system and in most peripheral organs. In contrast, SST28 is mainly produced by intestinal enteroendocrine cells and is the major isoform of the intestinal SST content [4]. SST mediates its biological effects via its five known (SST_1-5_) G-protein coupled receptors [2,5]. All of these receptors are coupled to Gα_i/o_, thus their activation leads to the suppression of adenylyl cyclase (AC) activity and a reduction in cellular adenosine 3’,5’-cyclic monophosphate (cAMP) levels, as well as an activation of phospholipase C (PLC) [2,5]. Since its discovery in 1973, seminal work has been performed to understand its role in various biological processes [6]. Its anti-secretory potential has been clinically exploited to treat endocrine diseases, such as acromegaly and neuroendocrine tumours [5]. Although a large quantity of information is available about its role in the action of endocrine regulation, inflammation and pain [7,8], little is known about its effects on the cardiovascular system, more specifically on endothelial barrier properties. Nevertheless, recently, SST, at least in part, via SST_1_ and SST_2_ receptors has been suggested to be involved in cardioprotection [9]. Both vasoconstrictive and vasodilative actions of SST have been reported in canine and rat vessels [10,11], and an improvement in flow-mediated vasodilation has been observed in acromegaly patients treated with SST analogues [10,11,12]. Moreover, SST and its analogues inhibit vascular smooth muscle cells (VSMCs), while promoting endothelial cell (EC) proliferation [13,14], thus reducing neointima formation [14]. Various human vessels express high levels of SST_1_ and low levels of SST_2_, SST_4_, and SST_5_ receptors [15,16]. However, the endothelial specific expression of SST receptors is not well documented. The activation of SST receptors leads to the activation of diverse signalling pathways depending on the tissue and type of receptors expressed. Since cAMP signalling plays an important role in the regulation of EC function [17,18], it is of interest to investigate SST effects on endothelial barrier integrity and angiogenesis.

The ECs form the single-layered inner lining of blood and lymphatic vessels [19]. There, they act as a semipermeable membrane and control the exchange of macromolecules, solutes, fluids, and cells, for example, leukocytes between blood and the extravascular space [20], thus maintaining tissue homeostasis. Additionally, the ECs secrete several vasoactive agents, which not only help to maintain the integrity of the endothelial barrier, but also regulate the platelet function and vascular smooth muscle tone, and thus actively participate in the regulation of blood pressure. Impaired EC barrier properties are hallmarks of several pathologies, such as acute lung injury, chronic inflammation and tumour progression [21]. The major regulators of EC barrier integrity include acto-myosin-based contractile machinery and adhesive molecules located at cell–cell and cell–matrix contacts [22]. The barrier properties of the endothelium may be altered by a variety of diverse circulating vasoactive agents and hormones, including somatostatin.

In the present study, we investigated the effects of SST (primarily SST14) on the EC barrier properties using a cell culture model of human umbilical cord endothelial cells (HUVECs). In the present paper, we report that SST primes the HUVEC monolayer for the agonist (thrombin)-induced hyperpermeability via an activation of the ras homolog family member A/Rho-associated coiled-coil kinase (RhoA/Rock) pathway. Furthermore, it primes the HUVECs for vascular endothelial growth factor (VEGF)-induced EC sprouting.

## 2. Results

### 2.1. HUVECs Mainly Express SST_1_ and SST_5_ Receptors

**Figure 1:**
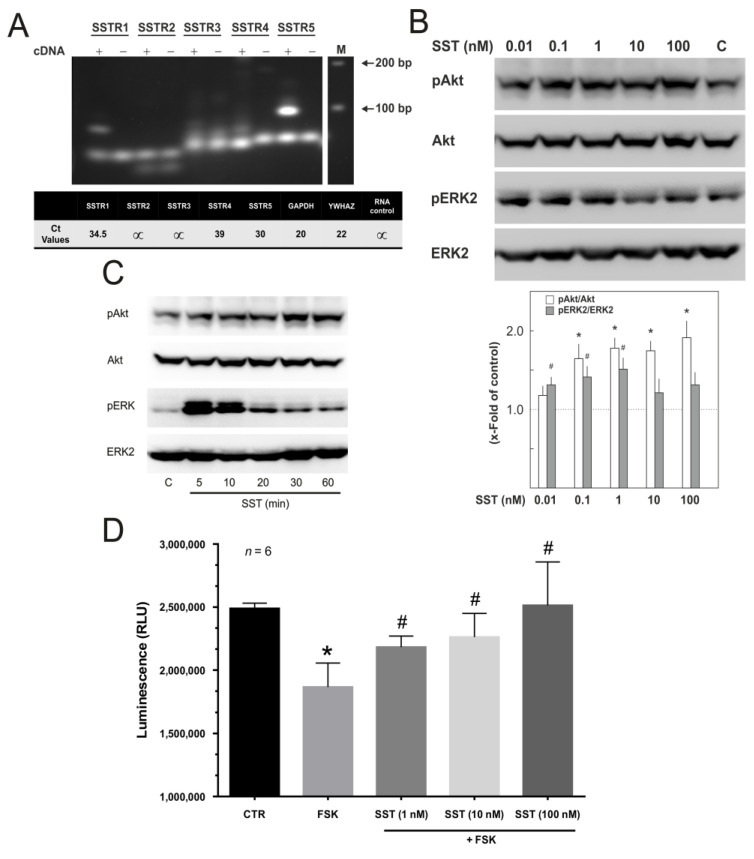
**SST receptor gene (SSTR) expression and signalling in HUVECs**. (**A**) The expression of SSTR mRNAs in HUVECs. The expected PCR fragment sizes for SSTR1-5 were 115 bp, 193 bp, 151 bp, 157 bp, and 94 bp, respectively. The plus (+) sign indicates cDNA and negative (-) sign indicates total RNA (without reverse transcriptase) used as the negative control. The lower panel shows Ct values determined by qPCR using HUVEC cDNA corresponding to 1 ng total RNA. (**B**) Concentration (as indicated)-dependent effect of SST14 on Akt and ERK2 phosphorylation. C: buffer-treated control. Representative Western blots of 3 experiments. The lower panel shows the quantification of the Western blots shown above, the dotted line shows the normalised basal levels of pAkt and pERK2. *n* = 3, *p* < 0.05; * vs. control (pAkt), ^#^ vs. control (pERK2). (**C**) Time-dependent effect of SST14 (1 nM) on Akt and ERK2 phosphorylation. Representative Western blots of phosphorylation and total Akt and ERK. (**D**) Concentration-dependent effect of SST14 (1 nM) on FSK-induced cAMP production. ECs were treated with SST14 or buffer (CTR) for 10 min followed by treatment with FSK (10 μM). *n* = 6, *p* < 0.05; * vs. control, ^#^ vs. FSK alone.

First, we determined the expression of SSTRs in primary HUVECs (passage 1) via PCR. As shown in Figure 1A, the HUVECs mainly express SSTR1 and SSTR5 mRNA. Further quantification by qPCR confirmed the expression of SSTR1 and SSTR5 with Ct values of 34 and 30, respectively. In order to verify the activation of SST receptor signalling, we analysed the phosphorylation state of mitogen-activated protein kinase (MAPK), extracellular signal regulated kinase (ERK2) and protein kinase B (Akt), and cAMP production in HUVECs. We used SST14 throughout our study. As shown in Figure 1B,C, the treatment of HUVECs with SST14 resulted in a concentration and time-dependent activation (phosphorylation on active sites) of both ERK2 and Akt. The maximum effect on Akt and ERK2 phosphorylation was observed at 1 nM concentration, which was not increased further at higher concentrations (Figure 1B). Even at the concentrations higher than 1 nM, a weaker effect on ERK2 phosphorylation was observed. SST14 induced a persistent Akt phosphorylation in HUVECs, observable even after 60 min of treatment. In contrast, ERK2 phosphorylation was transient reaching maximal in 5 min and then returning to a basal level within 30 min (Figure 1C). The relative quantification of cellular cAMP levels was performed using the cAMP-Glo^TM^ assay, where a reduction in the luminescence signal indicates an increase in cAMP levels. Forskolin (FSK), a direct activator of AC, was used to induce cAMP production. FSK caused a strong reduction in the luminescence signal (an indication of cAMP production), which was abrogated in HUVECs pre-treated with increasing concentrations of SST14 (Figure 1D).

### 2.2. SST14 Primed HUVEC Monolayers for Agonist-Induced Endothelial Hyperpermeability

**Figure 2:**
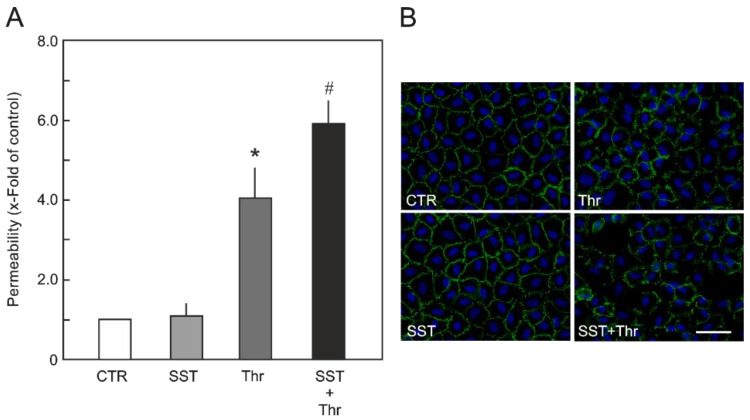
**Effect of SST on endothelial barrier function**. (**A**) HUVEC permeability: HUVEC monolayers cultured on filter membranes were treated with SST14 (1 nM) or buffer (CTR) followed by treatment with thrombin (Thr; 0.5 U /mL). Flux of albumin across HUVEC monolayers 10 min (maximum effect) after Thr treatment is presented. *n* = 5; *p* < 0.05; * vs. control, ^#^ vs. Thr alone. (**B**) HUVEC monolayers cultured on glass coverslips were treated with SST14 (1 nM) or buffer (CTR) followed by treatment with thrombin (Thr; 0.5 U /mL). Cells were fixed with ice-cold methanol and VE-cadherin was immuno-stained using a mouse anti-VE-cadherin (human) antibody. Scale bar: 50 μm.

In the next step, we investigated the effect of SST14 on the endothelial barrier function using an in vitro model of endothelial permeability [23]. In this model, we used thrombin as the agonist to transiently induce HUVEC hyperpermeability. As shown in Figure 2A, SST14 itself did not affect HUVEC permeability under basal conditions. Thrombin alone caused a 4-fold increase in the permeability of HUVECs, which was further increased to 6-fold in the HUVEC monolayers pre-treated with SST14 (1 nM). This SST14-mediated HUVEC barrier destabilizing effect was further verified by investigating the EC adherens junctions (AJs). For this, VE-cadherin (an important component of endothelial AJs) was immuno-stained using a specific antibody (Figure 2B). As observed in the case of permeability, the treatment of HUVECs with SST14 alone did not cause any changes in the localisation of VE-cadherin at the cell–cell junctions. The treatment with thrombin alone caused the displacement of VE-cadherin from the cell–cell junctions. This thrombin-induced effect was further amplified in the HUVEC monolayers pre-treated with SST14 (1 nM).

### 2.3. Role of Akt and MEK/ERK Pathways in SST14-Mediated Sensitisation of HUVEC Permeability

**Figure 3:**
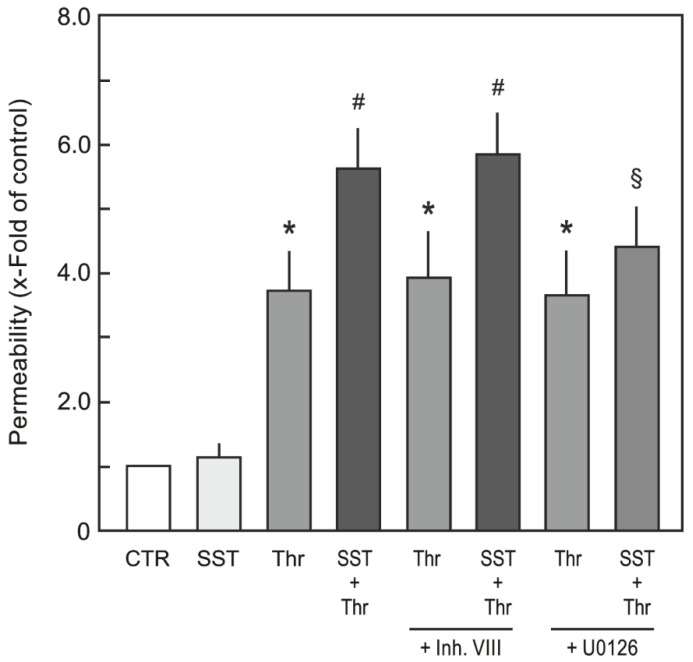
**Effect of inhibitors of Akt and MEK/ERK pathways on SST14-mediated HUVEC sensitisation**. HUVEC monolayers cultured on filter membranes were treated with SST14 (1 nM) or buffer (CTR) followed by treatment with thrombin (Thr; 0.5 U /mL). In the sets of experiments where Akt and MEK/ERK inhibitors were used, these were added 30 min before adding SST14 or buffer. Flux of albumin across HUVEC monolayers 10 min (maximum effect) after Thr treatment is presented. *n* = 3; *p* < 0.05; * vs. control, ^#^ vs. Thr alone, ^§^ vs. SST+Thr.

Since SST14 induced an activation of both PI3K/Akt and MEK/ERK signalling in HUVECs (Figure 1), we investigated the role of these pathways in the SST14-mediated sensitisation of HUVEC monolayer permeability. The activation of the PI3K/Akt and MEK/ERK pathways was pharmacologically inhibited using Akt inhibitor VIII and U0126, respectively. As shown in Figure 3, the pre-treatment of HUVEC monolayers with Akt inhibitor VIII did not abrogate SST14-mediated HUVEC sensitisation to thrombin-induced hyperpermeability. However, the treatment with an MEK/ERK inhibitor abrogated SST14-mediated HUVEC sensitisation to thrombin-induced hyperpermeability (Figure 3).

### 2.4. SST14 Primed Agonist-Induced Activation of RhoA/Rock Pathway

**Figure 4:**
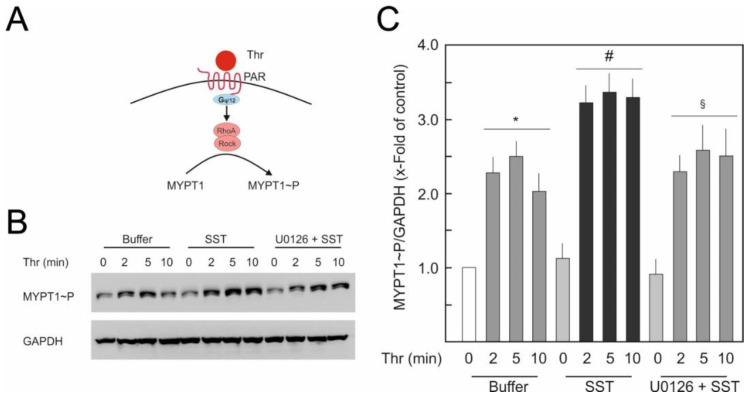
**Effect of SST14 on RhoA/Rock pathways in HUVECs**. (**A**) Schematic presentation of thrombin-mediated activation of RhoA/Rock signalling. (**B**) HUVECs were treated with thrombin (0.5 IU/mL) in the presence of buffer or SST14 (1 nM). Where indicated, cells were treated with U0126 (5 µM) for 30 min before treating with SST14 and thrombin. Representative Western blots of 3 experiments. (**C**) Quantification of blots from (**B**). *p* < 0.05; * vs. control, ^#^ vs. Thr alone, ^§^ vs. SST+Thr.

The RhoA/Rock pathway is one of the key mechanisms responsible for agonist-induced endothelial hyperpermeability. Therefore, we investigated whether SST14 exerts any effect on the activation of the RhoA/Rock pathway. The activation of the RhoA/Rock pathway was analysed by measuring the phosphorylation state of MYPT1 at Thr850, an endogenous direct target of Rock kinase. Figure 4A shows the schematic presentation of the thrombin-induced activation of RhoA/Rock signalling. As demonstrated in Figure 4B,C, thrombin induced MYPT1 phosphorylation in a time-dependant manner reaching maximum after 5 min. Although SST14 itself did not affect basal phosphorylation of MYPT1, it amplified the thrombin-induced MYPT1 phosphorylation, which was significantly attenuated in the presence of the MEK/ERK inhibitor U0126.

**Figure 5:**
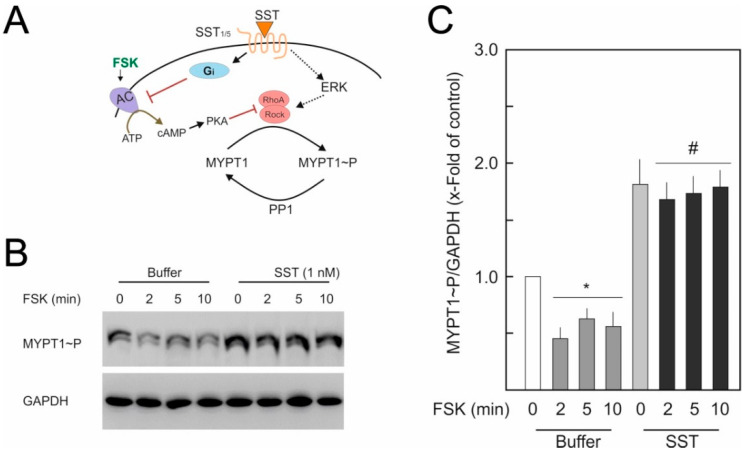
**Effect of SST14 on cAMP/PKA-mediated inhibition of RhoA/Rock signalling**. (**A**) Schematic presentation of FSK-mediated inhibition of RhoA/Rock signalling and the possible interaction with SSTR14 signalling. (**B**) HUVECs were treated with FSK (5 μM) in the presence of buffer or SST14 (1 nM). Where indicated, cells were treated with U0126 (5 µM) for 30 min before treating with SST and FSK. Representative Western blots of 3 experiments. (**C**) Quantification of blots from (**B**). *p* < 0.05; * vs. control, ^#^ vs. FSK alone.

The SST14-mediated sensitisation of the RhoA/Rock pathway is further verified by the manipulation of cAMP/PKA signalling. We previously demonstrated that the activation of cAMP/PKA signalling inhibits RhoA/Rock signalling [24]. Again, FSK, a direct activator of adenylyl cyclase, was used to activate the cAMP/PKA pathway and MYPT1 phosphorylation was used as an indicator of RhoA/Rock activity. Figure 5A shows the schematic presentation of FSK-mediated inhibition of RhoA/Rock signalling. As shown in Figure 5B,C, FSK induced the dephosphorylation of MYPT1 under basal conditions. SST14-treated cells show a higher basal MYPT1 phosphorylation. Moreover, the FSK-mediated reduction in basal MYPT1 phosphorylation is completely abolished in HUVECs pre-treated with SST14 (1 nM).

### 2.5. SST14 Moderately Increased HUVEC Proliferation and Primed HUVECs for VEGF-Mediated In Vitro Angiogenesis

**Figure 6:**
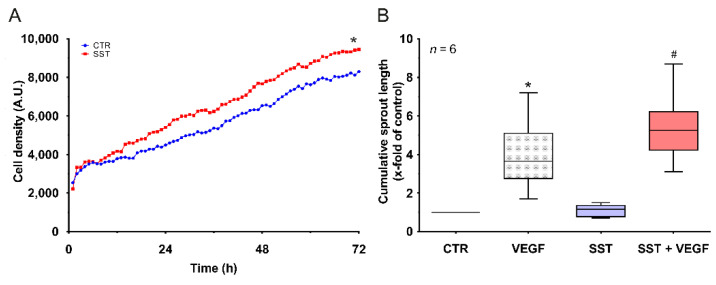
**Effect of SST14 on HUVEC proliferation and in vitro angiogenesis (3D Spheroids)**. (**A**) Cell density measured by time-lapse live-cell imaging. Cells were treated with 1 nM SST14 or buffer (CTR) under low-growth factor conditions and imaged after every 30 min for a period of 72 h. *n* = 3; *p* < 0.05; * vs. control. (**B**) 3D spheroid assay. HUVECs were treated with SST14 (1 nM) or buffer (CTR) followed by treatment with VEGF (10 ng/mL). *n* = 6; *p* < 0.05; * vs. control, ^#^ vs. VEGF alone.

Vessel renewal (angiogenesis) and the integrity of endothelial cell–cell junctions are two inter-linked physiological processes. Growth factor-mediated angiogenesis first results in the loosening of endothelial adherens junctions followed by cell migration, proliferation, and vessel sprouting. Since we observed the SST14-mediated priming of ECs towards agonist (thrombin)-induced hyperpermeability, we investigated whether SST14 also primes endothelial cells towards angiogenesis. Indeed, ECs cultured in the presence of SST14 showed a moderately higher proliferation rate when cultured under low growth-factor conditions (Figure 6A). Furthermore, angiogenesis was assessed by measuring the cumulative sprout length in a 3D spheroid assay (Figure 6B). Although SST14 itself did not increase basal sprouting, it primed ECs towards VEGF-mediated sprouting. HUVECs treated with SST14 demonstrated a higher cumulative sprout length in response to VEGF treatment compared to non-primed cells (VEGF alone).

## 3. Discussion

In the present study, we provide the first evidence that SST14 primes HUVECs for thrombin-induced hyperpermeability, at least partly via the MEK/ERK-mediated activation of RhoA/Rock signalling and promotes their proliferation leading to angiogenesis presumably by SST_1_ and/or SST_5_ receptor activation.

SST receptors are differentially expressed with overlapping or discrete distribution throughout the body. For example, SST_1_ and SST_2_ show a diffuse distribution in the whole brain, while SST_4_ receptors are highly expressed in the cortex, hippocampus, and habenula [25]. The endothelial expression of various SST receptor subtypes in various vascular beds is not well documented. Previously, it has been shown that endothelial-like EA.hy926 hybrid cell line express only SST_3_; bovine artery (undisclosed tissue) ECs express SST_1_, SST_3_, and SST_5_ [26], and human coronary artery ECs express SST_1_, SST_2_, and SST_5_ receptor mRNAs [27]. Another study [15] demonstrated that HUVECs (commercial, high passage) mainly express SST_1_ and low levels of SST_4_ receptor mRNA [15]. In partial agreement with this study, a previous study [28] showed that quiescent primary HUVECs (passage undisclosed) mainly express SST_1_, while proliferating HUVECs express SST_1_, SST_2_, and SST_5_ receptor mRNA [28]. The quiescence was defined as cells near to confluence and cultured without growth factors for 24 h, while proliferation was determined as cells doubling in number 24 h after seeding in the growth factors. Therefore, the effect of growth factors on SST receptor expression cannot be ruled out. In our study, primary HUVECs (passage 1) reaching 70% confluence, were cultured for another 24 h in reduced growth factor/serum medium to produce synchronised quiescent cells. Next, we demonstrated the expression of SST_1_ and SST_5_ mRNA, but did not observe SST_2_ mRNA.

We present the first data on SST14-induced Akt and/or MAPK phosphorylation in primary HUVECs. The activation of SST receptors leads to the induction of complex and various signalling cascades depending on the cell/tissue and type of the receptor(s) expressed. The inhibition of AC and the induction of protein tyrosine phosphatase (PTP) activities is common for all SST receptor types and are responsible for anti-secretory and pro-apoptosis effects of SST, respectively [29]. The anti-proliferative activity of SST and its analogues is mediated by modulating MAPK and Akt signalling pathways. The anti-proliferative actions are mainly linked to an inhibition of MAPK and Akt signalling [1,2,30,31]. However, several studies also demonstrate an activation of Akt and MAPK pathways. SST treatment of Chinese hamster ovary (CHO) cells stably expressing individual SST_1-4_ receptors induced ERK phosphorylation [32]. Accordingly, the treatment of HEK cells over-expressing individual fish (Epinephelus akaara) SST_1-4_ receptors with SST caused an increased ERK and Akt phosphorylation [33]. In accordance with these findings, cochlea from SST_1_ and SST_1/2_ double knockouts showed reduced basal Akt phosphorylation [34]. High concentrations of SST (1 μM) antagonised Kaposi’s sarcoma cells conditioned medium-induced ERK phosphorylation in EA.hy926 and BAEC [26]. In the present study, we observed a concentration- and time-dependent activation of both Akt and ERK2 phosphorylation, the optimal activation being observed at a 1 nM concentration, which is well below the quantity used in the above-reported studies (100 nM–1 μM). We observed a declined effect on ERK2 with higher concentrations. The activation of both Akt and ERK2 is associated with cell proliferation [35], EC survival and angiogenesis [36,37]. Accordingly, we observed an increased HUVEC proliferation rate/survival and VEGF-mediated in vitro angiogenesis. Our data is supported by a previous study [14], in which the suppression of vascular smooth muscle cells, but enhanced EC growth, were described in a rabbit aortic balloon injury model of neointima formation [14]. Likewise, an observational study reported a reduction in circulating endothelial progenitor cells in acromegalic patients, which was normalised after the treatment with SST analogue octreotide [38]. The anti-angiogenic effects of SST and its analogues are mainly observed in tumour angiogenesis, where SST_2_ and SST_3_ receptors are predominant [26,29,39]. Therefore, the differences between our data and previous studies may be explained by the absence of SST_2_ and SST_3_ receptors in our HUVECs in the applied culture conditions.

One of the main modulators of EC barrier integrity and cell–cell junctions is the RhoA/Rock signalling, and its hyper-activation leads to the disruption of EC barrier integrity [40,41]. Thrombin mediates its EC barrier disruptive effect mainly via the activation of RhoA/Rock pathway [40]. In the present study, the activation of the RhoA/Rock pathway was investigated by analysing the phosphorylation state of MYPT1, which is a direct endogenous substrate of Rock [42]. As expected, thrombin transiently induced MYPT1 phosphorylation, which was greatly enhanced in the presence of SST14. This suggests that SST14 primed RhoA activation, which was clearly observable under activated (thrombin) conditions. This priming effect was lost in the presence of the MEK/ERK inhibitor U0126. These set of data demonstrate the involvement of the MAPK/ERK pathway in the priming of RhoA activity in ECs. A direct activation of RhoA or Rock in ECs has not been yet reported. However, in COS-7 cells, the direct interaction of ERK with ectopically expressed RhoA-GFP, leading to increased RhoA activity was described [43]. The study demonstrates that this increased RhoA activity is due to ERK-mediated phosphorylation of RhoA at Ser188. The existence of this mechanism in ECs needs further investigations.

The cAMP/PKA signalling pathway acts antagonistically to RhoA/Rock signalling [24,44,45], and manoeuvres raising cellular cAMP levels suppress RhoA/Rock activity [44,46,47]. As all SST receptors are coupled with G_αi/o_ and their activation suppresses AC activity and reduces cellular cAMP levels (Figure 1D) [1], an upregulation of RhoA/Rock activity is expected. Indeed, an increased basal phosphorylation of MYPT1, a direct substrate of Rock, is observed (Figure 5B) in the present study, indicating an activation of the RhoA/Rock pathway. Moreover, FSK (an activator of AC)-mediated dephosphorylation of MYPT1 was lost in the presence of SST14, indicating AC inhibition. In contrast to our data, an early study showed that, in HUVECs, high concentrations of SST14 (100 nM) attenuate thrombin-induced RhoA activation, despite the strong inhibition of FSK-mediated AC activity and reduction in cellular cAMP levels [48]. The effect of SST14 on basal RhoA activity as well as the mechanism of RhoA inhibition was not investigated in that study. A plausible explanation may be an inhibition of thrombin-induced ERK activity, and hence downstream RhoA/Rock activity at higher SST concentrations.

In conclusion, this is the first demonstration that HUVECs under quiescence conditions express SST_1_ and SST_5_ receptors. SST14 treatment primes ECs for thrombin-induced hyperpermeability mainly via the activation of MEK/ERK signalling. Moreover, SST14 promotes HUVEC proliferation and in vitro angiogenesis. The detailed molecular mechanisms were not investigated in the present study, and further investigations are needed to exploit these endothelial effects of the SST receptor system for therapeutic use.

## 4. Materials and Methods

### 4.1. Materials

SST14 was from Bachem AG (Bubendorf Switzerland); anti-VE-cadherin antibody was from Beckman Coulter (Krefeld, Germany); the RT-PCR and qPCR reagents were from Bimake (Housten, TX, USA); anti-phospho MYPT1 (T850) and anti-GAPDH antibodies were from Cell Signaling Technologies (Danvers, USA); the Complete^®^ protease inhibitor cocktail was from Roche (Mannheim, Germany); ThinCert^®^ polycarbonate membrane filters (6-well) were from Greiner Bio-One (Frickenhausen, Germany); Benzonase^®^ was from Merck-Millipore (Darmstadt, Germany); the EC basal medium plus supplement pack was from PromoCell (Heidelberg, Germany); the HRP-conjugated anti-mouse IgG and -rabbit IgG antibodies were from Santa Cruz biotechnology (Heidelberg, Germany); human thrombin was from Sigma (Steinheim, Germany); Pierce^®^ ECL solutions were from Thermo Scientific (Darmstadt, Germany); and forskolin was from Tocris Bioscience (Bristol, U.K.). All other chemicals were of the best available quality, usually of an analytical grade.

### 4.2. Cell Culture

The study conforms to the principles outlined in the “Declaration of Helsinki” (*Cardiovascular Research* 1997; 35: 2–3). HUVECs were isolated from umbilical cords obtained from the gynaecology department at University hospital Giessen after approval from the ethics committee of the hospital and informed consent was obtained from the patients. The cells were cultured, as previously described [23], in a complete EC culture medium (Cat # C-22010; PromoCell, Heidelberg, Germany) and used at passages 1–2. HUVECs were cultured in 6-well plates for Western blotting, 6-well filter inserts or 8-well electrode arrays for permeability assays, and 10 cm culture dishes for the pulldown assay.

### 4.3. Experimental Protocols

The basal medium used in the incubations was modified Tyrode’s solution (composition in mM: 150 NaCl, 2.7 KCl, 1.2 KH_2_PO_4_, 1.2 MgSO_4_, 1.0 CaCl_2_, and 30.0 *N*-2-hydroxyethylpiperazine-*N*′-2-ethanesulfonic acid; pH 7.4, 37 °C). Agents were added as indicated. Stock solutions of SST14 and thrombin were prepared in water, U0126 and FSK in DMSO. Appropriate volumes of these solutions were added to the cells yielding final solvent concentrations ≤ 0.1% (vol/vol). Where the combination of drugs was used, inhibitors were added 30 min before adding the statins. The same final concentrations of water or DMSO were included in all respective control experiments.

### 4.4. Immunocytochemistry and Fluorescence Microscopy

Immunocytochemistry and confocal microscopy was performed, as previously described [17]. Briefly, HUVECs were grown until confluence on the glass cover slips. After treatment, the cells were fixed with methanol and blocked with blocking solution (5% BSA + 5% FCS) for 1 h. The cells were incubated with the primary antibody overnight at 4 °C and with the secondary antibody for 1 h at RT. The cover slips were embedded in fluorescent mounting medium (CitiFluor, U.K.) and put onto glass slides. Images were obtained using a Zeiss LSM 710 (Zeiss, Jena, Germany) confocal microscope.

### 4.5. Endothelial Monolayer Permeability

The permeability of trypan blue-labelled albumin across the HUVEC monolayers was analysed, as previously described [23].

### 4.6. Western Blotting

Western blotting was performed, as previously described [23]. The blots were imaged using Fusion-FX7 imager (VWR, Erlangen, Germany) and the unsaturated images were analysed using Quantity-One software (Bio-Rad, Feldkirchen, Germany). GAPDH from the same gel was used as a loading control for the normalisation of the respective protein signal.

### 4.7. Cell Proliferation Assay

The cell proliferation assay was performed by time-lapse live-cell imaging using the Juli^TM^ Br system (NanoEnTek Inc., Seoul, Korea).

### 4.8. cAMP-Glo^TM^ Assay

The cAMP-Glo^TM^ assay (Promega GmbH, Walldorf, Germany) was performed according to the manufacturer’s instructions. Briefly, the cells were cultured in 96-well white cell culture plates at the density of 5 × 10^3^ per well. After 24 h, the cells were treated with agonists/antagonists, as described in the respective figure legend, and the changes in cellular cAMP levels were analysed using the assay reagents. The luminescence was measured using a GloMax luminometer (Promega GmbH, Walldorf, Germany).

### 4.9. Statistical Analysis

The data are presented as the means (±S.E.M) of 3–5 experiments from independent cell preparations. The comparison between the two groups was performed by Student’s *t*-test and between multiple groups by one-way analysis of variance (ANOVA) followed by Tukey’s post hoc test using Graphpad Prism 6 software (Graphpad Inc., San Diego, CA, USA). The *p*-values of ≤ 0.05 were considered statistically significant.

## Data Availability

The data presented in this study are available within the manuscript.
